# A Crypto-Steganography Approach for Hiding Ransomware within HEVC Streams in Android IoT Devices

**DOI:** 10.3390/s22062281

**Published:** 2022-03-16

**Authors:** Iman Almomani, Aala Alkhayer, Walid El-Shafai

**Affiliations:** 1 Security Engineering Lab, Computer Science Department, Prince Sultan University, Riyadh 11586, Saudi Arabia; akhayer@psu.edu.sa (A.A.); or walid.elshafai@el-eng.menofia.edu.eg (W.E.-S.); 2 Computer Science Department, King Abdullah II School of Information Technology, The University of Jordan, Amman 11942, Jordan; 3 Department of Electronics and Electrical Communications Engineering, Faculty of Electronic Engineering, Menoufia University, Menouf 32952, Egypt

**Keywords:** ransomware hiding, multimedia steganography, encryption, embedding, IoT, HEVC, AES, LSB, quality assessment, multi-level security, antivirus, confidentiality and integrity

## Abstract

Steganography is a vital security approach that hides any secret content within ordinary data, such as multimedia. This hiding aims to achieve the confidentiality of the IoT secret data; whether it is benign or malicious (e.g., ransomware) and for defensive or offensive purposes. This paper introduces a hybrid crypto-steganography approach for ransomware hiding within high-resolution video frames. This proposed approach is based on hybridizing an AES (advanced encryption standard) algorithm and LSB (least significant bit) steganography process. Initially, AES encrypts the secret Android ransomware data, and then LSB embeds it based on random selection criteria for the cover video pixels. This research examined broad objective and subjective quality assessment metrics to evaluate the performance of the proposed hybrid approach. We used different sizes of ransomware samples and different resolutions of HEVC (high-efficiency video coding) frames to conduct simulation experiments and comparison studies. The assessment results prove the superior efficiency of the introduced hybrid crypto-steganography approach compared to other existing steganography approaches in terms of (a) achieving the integrity of the secret ransomware data, (b) ensuring higher imperceptibility of stego video frames, (3) introducing a multi-level security approach using the AES encryption in addition to the LSB steganography, (4) performing randomness embedding based on RPS (random pixel selection) for concealing secret ransomware bits, (5) succeeding in fully extracting the ransomware data at the receiver side, (6) obtaining strong subjective and objective qualities for all tested evaluation metrics, (7) embedding different sizes of secret data at the same time within the video frame, and finally (8) passing the security scanning tests of 70 antivirus engines without detecting the existence of the embedded ransomware.

## 1. Introduction

The revolution of IoT systems has been growing significantly through the development of daily life applications in various fields, including smart cities, education, healthcare, and considerably more. IoT systems primarily consist of sensors and electronic devices, which collect data and perform required actions accordingly [1]. Many advanced IoT devices, specifically smartphones and smartwatches, are built on the Android platform due to its high resilience and hardware support [2]. These Android-based IoT devices provide the user a convenient way to interact and control other IoT devices such as smart TV, cameras, and refrigerator through special applications. Thus, the Android operating system (OS) is essential for communication and data exchange in IoT devices. However, the continuous emergence of Android IoT devices increases malicious attacks. In 2019, the total Android malicious software detected globally reached 10.5 million, whereas the malware development increased by 480,000 in 2020 (https://www.statista.com/statistics/680705/global-android-malware-volume/ accessed on 1 September 2021). Such malicious attacks may trespass the victim device to affect other connected nodes on the network, resulting in violating the whole IoT ecosystem.

Among the most notorious harmful malware that threatens Android devices is ransomware. Ransomware is a subset of malicious software which takes control over the victim’s device by blocking access to the system resource or encrypting sensitive data. The victim gets the control back only if a ransom is paid to the attacker. According to [3], ransomware targets individual users, governmental parties, and business companies resulting in significant financial loss. Mainly, ransomware is classified into two main categories, locker-based ransomware, and crypto-based ransomware. In the locker-based ransomware, a lock screen is displayed on the user device blocking access to the system resources. Hence, a ransom is requested to remove the lock window [4]. On the other hand, the crypto-based ransomware encodes the system data requesting a ransom to retrieve the decryption key.

Even though several works have been proposed on implementing ransomware detection systems [5,6,7,8], studies have discussed that such systems might not be sufficient enough in detecting the malicious software that implements steganography and encryption techniques [9]. Steganography is the art of concealing the presence of secret data by embedding it within a cover object [10]. The applied techniques of steganography are mainly classified into three categories based on how covert communication is established [9]. In the first technique, the malicious software is concealed by simulating the network traffic of a benign app. While in other types of steganography methods, parts of the malicious software components are embedded within the network traffic. However, in media steganography techniques, the malicious software is embedded within a media file. Among several media files that can be utilized as a carrier object, the high-efficiency video coding (HEVC) is gaining increasing interest due to its high embedding capacity [11,12].

Additionally, attackers might combine the implementation of steganography algorithms with encryption techniques to increase the undetectability levels of malicious software. In this case, even if a detection system revealed the hidden ransomware application, it will not be classified as malware due to the implemented encryption technique. However, in the scope of IoT devices, the limited resources and restricted energy consumption means that the encryption algorithm must be carefully selected and implemented in IoT-based solutions [13]. Consequently, the authors in [14] compared current encryption algorithms such as AES, RSA, RC6, 3DES, and blowfish regarding energy consumption. The Advanced Encryption Standard (AES) encryption algorithm is widely utilized by IoT security solutions due to its low power consumption [15,16,17] compared to other ciphers, especially asymmetric ciphers like RSA. AES is suitable when security and complexity concern the applied security solution. AES is a symmetric encryption algorithm used with a minimum 128-bit key and 128-bit initial value (IV) [18]. Symmetry indicates that for both encryption and decryption process, the same cipher key is used.

In this work, an offensive algorithm is presented in which the ransomware is firstly encrypted and then concealed within HEVC video streams. The main contributions of this paper are listed below:

(a) Study the related state-of-the-art research to examine any introduced efforts to conceal ransomware apps within video streams.
(b) Summarize the recent literature approaches employing steganography algorithms in concealing malicious software within multimedia data.
(c) Propose a novel, efficient hybrid crypto-steganography approach to conceal encrypted ransomware apps using AES encryption and LSB (least significant bit)-based HEVC steganography.
(d) Achieve randomness embedding for a proper selection of cover media pixels for hiding secret ransomware bits.
(e) Employ objective and subjective simulations in detail to comprehensively assess the proposed multi-level security approach.
(f) Achieve superior performance in the objective-based results for all examined 19 assessment metrics used to evaluate the HEVC stream quality after ransomware embedding, compared to recent related works.
(g) Obtain extraordinary similarity in the subjective-based results between the original HEVC stream and stego HEVC stream resulting after ransomware embedding.
(h) Succeed in bypassing 70 famous antivirus engines as part of the security analysis for the concealed ransomware within the HEVC stream.
(i) Extract secret ransomware apps successfully with high performance.

The primary threat model presented in this research exploits that most of the existing detection approaches take ransomware as an input to their systems. They assume that ransomware is always a visible app that can be scanned through their prediction systems. Even if the victim’s detection system can detect the existence of the hidden ransomware, assuming they have the original cover video, it still needs to extract it. If the system manages to extract the hidden ransomware, it will find it encrypted, and the system requires the key to decrypt it, which is also challenging. This paper’s main contribution is to bring attention to the ransomware detectors from an offensive point of view that ransomware could be distributed using cryptography & steganography. Cryptography encrypts the ransomware to ensure that even if the steganography approach is broken (in case the detectors know the steganography approach and original cover video), the detectors still cannot recognize that this app is ransomware.

The rest of the paper is structured as follows. Section 2 presents a literature review on the related work on ransomware detection and hiding techniques in the IoT devices that were recently investigated. Section 3 discusses the proposed crypto-steganography approach. Section 4 provides the results analysis and discussion. Finally, the paper is concluded and future work is presented in Section 5.

## 2. Literature Review

Due to the explosive expansion of IoT systems, the number of connected nodes is increasing dramatically. However, from this current IoT era and its related applications have arisen several security attacks including denial of service (DoS) and malware control which alarms real threats for any IoT device [19]. To overcome the main vulnerabilities of Android IoT devices, several solutions have been proposed to detect IoT Android malicious software using machine learning [1,20], neural network [2], and deep learning [21].

In [2], the authors developed a multi-layer deep learning model that provides decisions via analyzing the previous malware features. Initially, the features of the malicious software were divided into clusters. Subsequently, the detection system applied a unique deep learning model on each separated feature set. Finally, it combined the results in order to generate the final decision. Furthermore, they have stored the resulting decisions in a blockchain to be further utilized in training specialized clusters. Another IoT malware detection approach was proposed by [1]. The proposed scheme conducted a vulnerability prediction analysis on Android IoT applications by implementing machine learning algorithms. Even though the system provided recommendations on reducing the risk vulnerabilities, it lacks large dataset samples that might affect the performance of machine learning algorithms that generate the security measures. The authors of [21] implemented a deep learning approach to develop two malware detection algorithms. The proposed methods consume few resources; hence they were sufficient to be used by Android IoT devices.

Kumar et al. proposed a run-time permission-based IoT malware detection scheme [22]. Initially, the detection algorithm performed a permission ranking and feature selection based on the permission similarity. Then, it applied permission mining utilizing association rules. Besides employing permissions in developing the IoT malware detection system, the authors of [23] utilized the application programming interface (API) calls to develop an event-driven detection system. By creating vent groups that describe behaviors of the application, the proposed system was able to classify new types of malicious software. Another testing framework was proposed by [24] which also aims to perform security analysis on IoT devices for new malware families using machine learning algorithms. Furthermore, the proposed test-learning framework implements genetic algorithms to generate Android malware applications for IoT devices.

The authors of [25] developed an IoT malware detection framework that combines several machine learning algorithms along with the advanced technology of blockchain. The proposed framework extracted the malicious information and fed it to the blockchain by utilizing a classification algorithm, and consequently, improving the efficiency of the malware classification process since all the malicious information is stored in the blockchain. An additional proposed framework that uses the machine learning approach was proposed by [26]. The authors developed a subset of features based on examining the performance of various feature selection algorithms. However, the machine learning approach can be further utilized in detecting encrypted malware. Azmoodeh et al. presented a ransomware detection algorithm that monitors the power consumption of Android IoT devices by implementing machine learning algorithms [27]. The system classifies the ransomware by calculating the energy consumption of the running processes.

Despite the advanced proposed solutions for IoT malware detection, malware steganography is still considered a challenge that requires further investigation [9]. In [28], the authors investigated the effect of embedding malicious software within a media file. Initially, they embedded a Metasploit malware within an image. After that, they performed two detection scenarios before and after implementing the steganography algorithm using Open Source Intelligence Tools such as VirusTotal. However, the results showed that several anti-virus engines could not detect the hidden malware. Nevertheless, the hidden malware was detected by some other anti-virus scanners. Another malware steganography attach was presented by [29]. In the proposed steganography system, the identity of the malware was hidden by generating fake network traffic, which mimics benign application traffic. Furthermore, steganography techniques can be utilized to create covert communication channels between the attacker and the malware. In [30], the authors developed a covert channel using the StegBlocks technique, which enables text communication between the active malware and its developer. However, the communication text file is limited to 23 KB.

In several research works, stenography techniques are combined with other cryptography algorithms to enhance the security and privacy of hidden secret data. In [31], the authors combined cryptography and stenography techniques to obtain a high security standard. Initially, the data was encrypted using an AES algorithm. Subsequently, the resulting encrypted data was concealed in 3D cover images. Arraziqi et al. have also employed the AES algorithm as an additional security layer to their proposed stenography scheme [32]. Furthermore, the authors of [33] have also adopted the AES algorithm as a security technique to encrypt the data before hiding it with the carrier file. However, in [31], two schemes were proposed in which LSB stenography is applied along with image processing and cryptography in order to secure sensitive data. Moreover, the authors of [34] have proposed a new embedding algorithm in which they apply sequential multiples for LSB real-time data hiding algorithm. Additionally, they apply a visual cryptography algorithm for security robustness.

In IoT platforms, a successful ransomware attack does not just result in compromising the victim’s personal data, but also leads to hijacking the device functionality. Thus, ransomware can be considered a devastating threat with consequences of compromising billions of interconnected IoT devices. Frantic Locker is an example of a locker ransomware that hijacks smart TVs which blocks the display screen and disables the reset option. The ransomware is activated when the user runs a malicious movie application. Frantic Locker requests a ransom of $500 USD to be paid within three days [35]. In the research field, some ransomware applications have been developed to demonstrate the threat ransomware can cause to the IoT networks. Android Simplocker is locker ransomware developed by Symantec researchers [36]. The proposed ransomware penetrates the Android wearable device once the device is connected to a smartphone. Consequently, Android Simplocker locks the display of the wearable device. A proof of concept was proposed by Nassi et al. [37] to infiltrate the damage of ransomware on IoT devices in business organizations. A smart bulb was hijacked by utilizing an Android smartphone from a close car. The proposed attack proofs that ransomware can compromise and control the entire IoT network.

Based on the above discussion, it can be concluded that most of the proposed detection systems assume that the ransomware is visible to be analyzed. Furthermore, the related works lack investigating the threat of encrypted malicious software. Therefore, in this paper, a novel offensive algorithm is proposed in which malware in general, and ransomware specifically, is encrypted and hidden in media streams.

## 3. Proposed Crypto-Steganography Approach

This section introduces the crypto-steganography approach that composes three main processes: AES encryption, random pixel selection process, and LSB-based steganography. The major advantage of employing the LSB-based steganography process is achieving high imperceptibility. This is why recent works [38,39] have utilized LSB to hide benign data.

LSB steganography can hide the secret ransomware bits inside the LSBs of the cover video frames without causing a noticeable difference between the cover and stego frames. Therefore, by altering the LSBs of the color components of the input cover video frame to a particular target binary value, the pixel’s color is slightly changed without making discrepancies in the resulting stego frame that human eyes can recognize.

To achieve and develop a secure and robust ransomware hiding within HEVC frames, both symmetric AES encryption and random pixel embedding processes are implemented during the LSB steganography process. The associated secret key employed in the ransomware embedding process must be the same in the ransomware extraction process. In contrast, the embedding bits in the video cover frame are changed randomly during the hiding process. Therefore, without knowing and utilizing the original secret key, it is challenging and not possible to recover and retrieve the concealed ransomware data in the video frame. Thus, the decoder side needs to know the right secret key to rescue the hidden ransomware data within the stego video frame.

**Figure 1:**
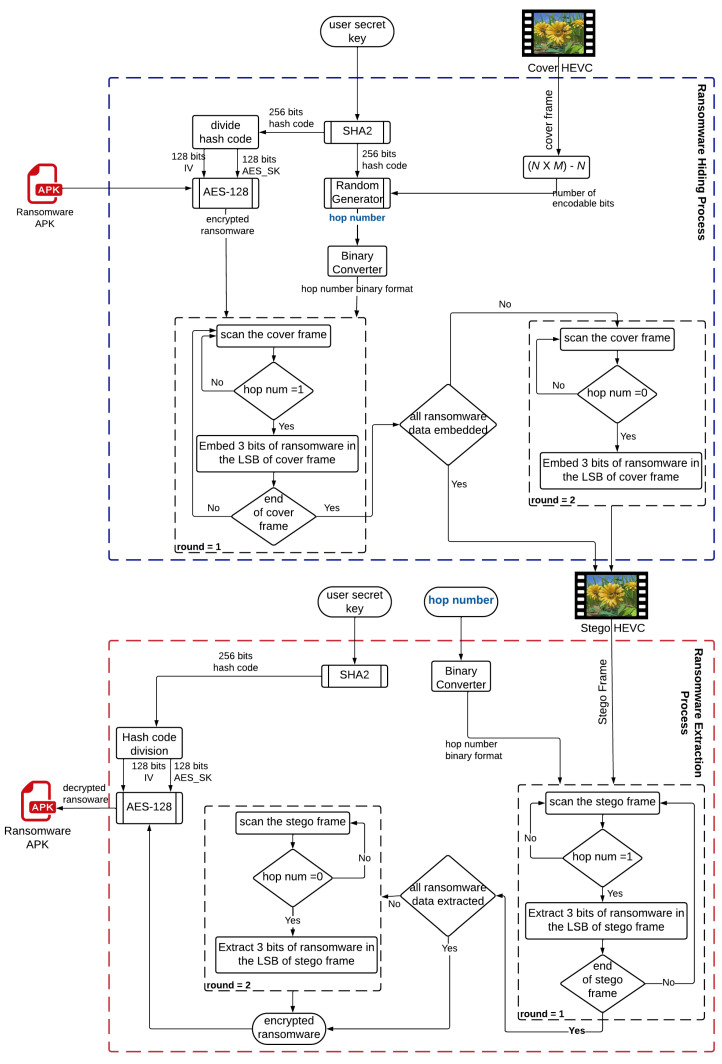
Proposed hiding/extraction stages of the crypto-steganography approach.

The proposed crypto-steganography approach is established based on the introduced image steganography algorithm presented in [40]. The crypto-steganography approach comprises two algorithms; Algorithm 1: the ransomware hiding process, and Algorithm 2: the ransomware extraction process as indicated in Figure 1. Firstly, a user-defined secret key is used to control the random pixel embedding process to hide the encrypted ransomware in the cover video frame. Additionally, this secret key is used to generate the initial parameters to run the AES encryption algorithm and control the operation of the employed random number generator (RNG). The secret encrypted ransomware bits are randomly concealed within the HEVC frame to preclude the non-dedicated and undefined decoders from identifying the concealed secret data. One of the main contributions of the crypto-steganography approach is its capability to hide any size and amount of ransomware samples within the HEVC frame, constrained merely by the cover video frame size.

The ransomware hiding algorithm takes as an input the cover HEVC frame (*cover_frame*), the user-defined secret key (*user_SK*), and the ransomware sample. Initially, the *user_SK* is forwarded to a hashing algorithm (SHA-256) to produce a secret hash code (*hash_code*) with 256 bits. The obtained *hash_code* is utilized to control the employed AES-128 encryption algorithm as follows:

(a) The first part of the generated hash code (128 bits) is used as an initialization vector (*IV*) for the applied AES-128 encryption algorithm.
(b) The second part of the generated hash code (128 bits) is used as a symmetric secret key (*ASE_SK*) to encrypt the ransomware using AES-128 encryption algorithm.

**Algorithm 1** Ransomware Hiding Process.1:**procedure**Ransomware_Hiding2:    **Input:** cover_frame, user_SK, ransomware3:    **Output:** stego_frame4:*Initialisation*:5:    Initialize hash_code: 256 bits6:    Initialize IV: 128 bits7:    Initialize AES_SK: 128 bits8:    hash_code = **SHA2** (user_SK)9:    IV = First_Part (hash_code)10:    AES_SK = Second_Part (hash_code)11:    encrypted_RW=**AES-128** (ransomware, IV, AES_SK)12:    num_encodable_bits= (M×N) – *N*13:    First_Part (cover_frame, R0) = encrypted_RW.length14:    hop_num = **RNG** (num_encodable_bits, hash_code)15:    hop_binary = Convert_Binary (hop_num)16:    round = 117:    cover_frame_modified = cover_frame18:    **while** (encrypted_RW.data ≠ 0) **do**19:        **if** (hop_binary.pixel = 1) and (round = 1) **then**20:           cover_frame_modified.LSB = **Embed** (encrypted_RW, 3 bits)21:        **end if**22:        **if** (hop_binary.pixel = 0) and (round = 2) **then**23:           cover_frame_modified.LSB = **Embed** (encrypted_RW, 3 bits)24:        **end if**25:        **if** (cover_frame.end = True) **then**26:           round = 227:        **end if**28:    **end while**29:    setgo_frame = cover_frame_modified30:**end procedure**

Furthermore, the whole generated hash code (256 bits) is utilized as an input to the random number generator (*RNG*) to generate the hop number (*hop_num*). The RNG further requires as an input the total number of encodable pixels (*num_encodable_bits*). Hence, this is calculated from the cover video frame ((*M*×*N*)-*N*), where *M* and *N* are the dimensions of the cover video frame. The generated hop number (*hop_num*) has the same binary size as the total number of encodable pixels, where each one of the encodable pixels requires to be characterized by a one-bit of the generated random hop number. It is noticed that the total encodable pixels are calculated by subtracting the length by one row from the video frame size (*M*×*N*), where this first row (row 0) is reserved to be the video frame header. This reserved video frame header has a length of 10 pixels that encompasses the length of the encrypted ransomware data in binary. The length of the encrypted ransomware data is an essential parameter to be stored within the video frame. The steganography approach exploits this length to decide where to finish the embedding and extraction processes.

Prior to starting the embedding step, the generated random hop number (*hop_num*) is converted into a binary sequence format (*hop_binary*). Consequently, this binary sequence is considered as the prominent secret control key for randomly hiding the encrypted ransomware data within the HEVC frame. The embedding step starts from the first pixel of row 1 of the cover HEVC frame (*cover_frame*). The embedding step is repeated for several rounds until all the data of the encrypted ransomware (*encrypted_RW*) is fully concealed in the cover frame. For the first round, three bits of encrypted ransomware data (*encrypted_RW*) are embedded in each LSB pixel of the colored HEVC frame only if this pixel maps to 1 in the random binary sequence. Nevertheless, in this initial round, the pixel is skipped if the pixel maps to 0 in the random binary sequence.
**Algorithm 2** Ransomware Extraction Process.1:**procedure**Ransomware_Extraction2:    **Input:** stego_frame, user_SK, hop_num3:    **Output:** ransomware, original_frame4:*Initialisation*:5:    Initialize hash_code: 256 bits6:    Initialize IV: 128 bits7:    Initialize AES_SK: 128 bits8:    Initialize decrypted_file_length: 10 pixels9:    hash_code = **SHA2** (user_SK)10:    IV = First_Part (hash_code)11:    AES_SK = Second_Part (hash_code)12:    num_decodable_bits = (*M*×*N*) – *N*13:    decrypted_file_length = First_Part (cover_frame, R0)14:    hop_binary = Convert_Binary (hop_num)15:    round = 116:    **while** (decrypted_file_length ≠ 0) **do**17:        **if** (hop_binary.pixel = 1) and (round = 1) **then**18:           encrypted_RW = **Extract** (stego_frame, 3 bits)19:        **end if**20:        **if** (hop_binary.pixel = 0) and (round = 2) **then**21:           encrypted_RW = **Extract** (stego_frame, 3 bits)22:        **end if**23:        **if** (stego_frame.end = True) **then**24:           round = 225:        **end if**26:    **end while**27:    ransomware = **AES-128** (encrypted_RW, IV, AES_SK)28:**end procedure**

However, if the end of the cover HEVC frame is reached while there is still encrypted ransomware data that need to be embedded, the embedding step is repeated for a second round. Starting from row 1 of the cover HEVC frame, the residual pixels in the encrypted ransomware data are embedded in the pixels of the cover HEVC that were initially skipped. Therefore, each pixel of the cover video frame maps to 0 in the random binary sequence is currently embedded with the residual of encrypted ransomware secret data. Finally, the stego video frame is obtained by collecting the encoded pixels resulted from the embedding process.

It is demonstrated from the steps of Algorithm 1 that the whole pixels of the cover HEVC frame are potentially exploited for embedding the encrypted ransomware data as desirable based on its length. Furthermore, the most crucial advantage is that the encrypted ransomware data is widely spread throughout the cover video frame, which is considered a type of robustness and security against intruders. This is because by checking the LSBs of the pixels within the stego video frame, it is difficult to differentiate or guess whether or not these pixels have been modified or not. This is due to ciphering and scrambling the secret ransomware data utilizing the AES algorithm. So, the original LSBs of the pixels within the cover video frame may have identical values to the encrypted bits of the secret ransomware data being hidden. Consequently, no alteration would be observed in the resulting stego video compared to the original video frame.

Algorithm 2 presents the ransomware extraction process that takes as an input the stego HEVC frame (*stego_frame*), user-defined secret key (*user_SK*), and the generated hop number (*hop_num*). The extraction process implements the same steps described in the embedding process but in a reverse manner. Initially, all the required credentials (*IV* and *AES_SK*) are generated to be further used in the AES decryption algorithm. Then, the encrypted ransomware data is extracted from the cover video frame. Finally, the accumulated encrypted ransomware data is decrypted using AES algorithm to get the original ransomware data.

## 4. Results and Comparisons

This section validates the proposed crypto-steganography approach by testing its performance on different resolutions of HEVC frames (cover media) and different sizes of ransomware files (secret data) to assess its hiding and extracting efficacy. This demonstrates the superiority of the proposed approach in hiding and extraction competence in the case of using different ransomware sizes within cover frames while preserving the video frame imperceptibility without affecting its visual quality and without recognizing the presence of ransomware data. Table 1 and Table 2 show the examined stream resolutions and ransomware sizes, respectively. We comprehensively utilized subjective and objective assessment metrics to evaluate the proposed crypto-steganography approach.

For subjective evaluations, the visual results in terms of the cover/stego frames are presented, in addition to the difference between them as will be demonstrated in Section 4.1. Moreover, the visual outcomes of the histograms and Gaussian Laplacian edges of the cover and stego frames are offered in Section 4.2. Furthermore, for objective evaluations, more and different 19 quality assessment metrics that are listed in Table 3 with their optimal values were utilized to test the efficacy of the proposed approach.

### 4.1. Visual Results Analysis

Visual analysis is used to check the amount of modification resulting in the stego frames after hiding the ransomware within the cover frames. Table 4 presents the visual outcomes of the examined cover and stego frames in case of utilizing various sizes of secret ransomware data. Furthermore, Table 4 presents the acquired difference between the stego and the cover frames (Entropy). From the obtained outcomes, it is noticed that the proposed crypto-steganography approach successes in hiding more ransomware samples (different sizes) inside the cover media frames (different resolutions) while preserving the perceptual quality (imperceptibility). Besides, the similarity assessment between the stego and cover frames can be evaluated using the estimation of the bit error rate (BER). BER estimates the error rate in the pixel bits between the stego and cover frames, which is represented in Equation (1) [41]:

(1) $$\mathrm{BER}=\frac{\sum_{m=1}^{M}\sum_{n=1}^{N}\left|x\left(m,n\right)-x^{\prime }\left(m,n\right)\right|}{M\times N}$$

where *x*(*m*, *n*) refers to the cover frame, $x^{\prime }$(*m*, *n*) refers to the stego frame, and *M* and *N* are the width and height of the video frame, respectively. Thus, the BER metric can be determined as the actual number of bit positions that are modified in the stego video frame compared to the cover video frame. It is recommended to achieve lower BER for accomplishing higher steganography performance. The results reveal that the stego frames were completely similar to the cover frames by obtaining zero BER values. Additionally, the diminutive difference in the entropy values for all examined HEVC streams proves the proposed steganography approach’s high concealing performance and efficacy.

Additionally, we calculated the computational CPU time of the proposed approach as indicated in Table 4 (Column 2, next to the Entropy value). The results confirm that the proposed stego-cryptography approach has satisfied complexity in embedding the encrypted ransomware file inside a video frame. This also shows the complexity of the extraction process, which is an identical inverse of the embedding process.

### 4.2. Histogram and Edge Results Analysis

Histogram analysis shows a relationship between the pixel frequency and its intensity value of the video frame. Table 5 presents the outcomes of the pixel intensity distributions (histograms) of the stego/cover video frames introduced in Table 4. These obtained histograms substantiate and verify the high-performance accomplishment of the proposed approach, where both stego/cover video frames have roughly identical pixel distributions. Furthermore, the acquired histograms of the difference frames between stego/cover frames are around the zero value, thus there are no pixel distributions for them. To further verify the efficiency of the proposed approach, the visual outcomes of the Gaussian Laplacian edges are introduced as shown in Table 6. These Gaussian Laplacian edges can be obtained by estimating the value of the Laplacian operator for the whole pixels within the stego and cover video frames. The Laplacian operator can be represented by *L*(*x*(*m*, *n*)), where *x*(*m*, *n*) is the input stego or cover video frame, and (*m*, *n*) refers to the spatial location of the video frame pixels. This operator can be mathematically represented as in Equation (2) [56].

(2) L(x(m,n))=x(m+1,n)+x(m−1,n)+x(m,n+1)+x(m,n−1)−4x(m,n)

Furthermore, the edge similarity between the stego and cover frames can be evaluated using the calculation of edge differential ratio (*EDR*). *EDR* estimates the pixel error rate in edges between the stego and cover frames. Therefore, a low *EDR* value is recommended. It is represented in Equation (3) [57].

(3) $$EDR=\frac{\sum_{m,n=1}^{K}\left|B\left(m,n\right)-\bar{B}\left(m,n\right)\right|}{\sum_{m,n=1}^{K}\left|B\left(m,n\right)+\bar{B}\left(m,n\right)\right|}$$

where the binary pixel value of the cover frame edges is estimated by *B*(*m*, *n*), and the related binary pixel value of the setgo frame edges is estimated by $\bar{B}$(*m*, *n*). It appears from the results shown in Table 6 that both stego/cover video frames have identical edge distributions for their whole included objects by achieving zero EDR values for all examined HEVC streams. This verifies the hiding efficiency of the proposed approach for maintaining the same edge distributions of the stego frames compared to the cover frames.

In addition, a comparative analysis study is conducted to compare the proposed crypto-steganography approach with recent steganography approaches as presented in Section 4.3. This study employed 17 objective-based metrics to apply fair and deep comparison with the related works.

From a security perspective, the encrypted ransomware embedding performance is validated using antivirus scan analysis as indicated in Section 4.4. Seventy well-known antivirus engines were used to scan the ransomware before embedding and after embedding (stego frame & infected video). Finally, the integrity of the hidden ransomware was examined and proved in Section 4.5.

### 4.3. Objective Quality Assessment Analysis

To further ensure the superiority of the proposed crypto-steganography approach, we compared its accomplishment with recent related steganography algorithms that are introduced in [38,39,58]. These introduced algorithms are based on only one stage of LSB steganography without employing further security stages like encryption or random embedding strategy as investigated in our proposed work in this paper. Table 7 shows the objective outcomes of the comparative analysis between the proposed approach compared to the related algorithms [38,39,58] in terms of all quality assessment metrics that were presented in Table 3.

As elaborated in Table 3, some of these objective quality metrics must have lower values, and others must have higher values to achieve the recommended and appreciated performance for the proposed crypto-steganography approach. In this perspective, it can be concluded from Table 7 that our proposed crypto-steganography approach outperforms almost all of the objective quality assessment metrics in comparison with the related works. This is obvious by obtaining lower values of the MSE, NAE, AD, LMSE, MD, SC, and ΔE metrics and higher values of PSNR, NK, SSIM, FSIM, MS-SSIM, UQI, SNR, NQM, WSNR, and VIFP metrics for the proposed approach compared to the related steganography approaches.

Therefore, the proposed crypto-steganography approach is highly recommended for ransomware hiding applications due to: (a) ensuring ransomware data integrity, (b) achieving high imperceptibility, (c) adding an extra level of security by applying AES encryption incorporated with steganography algorithm, (d) the capability of randomness in ransomware embedding process (e), accurate ransomware extraction, and (f) the capability of embedding different sizes of secret data within various video streams with different features.

### 4.4. Ransomware Security Scanning Analysis

As part of the security test, ransomware scanning analysis was performed utilizing the VirusTotal platform (https://www.virustotal.com/ accessed on 1 August 2021). VirusTotal is an online detection system that provides scanning services for any data type. VirusTotal utilizes over 70 anti-virus engines to perform malware detection and classification services [59]. Moreover, to ease the scanning process for large-scale databases, VirusTotal provides an API service in which researchers can upload the entire dataset and perform the scanning process without using a web interface. As a result, business companies have widely used the VirusTotal platform to diagnose their products’ false negatives and positives. Furthermore, its scanning services have been heavily used by researchers in the security field to detect several malware families [60].

**Figure 2:**
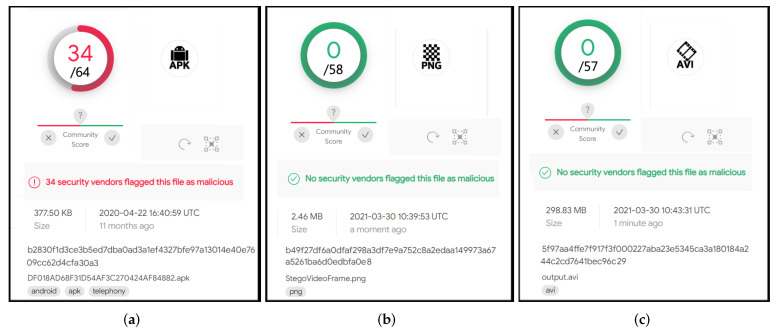
VirusTotal scanning results. (**a**) Ransomware scan result. (**b**) Stego frame scan result. (**c**) Infected video scan result.

In our experiment, ransomware scanning analysis was implemented on three different files. Before the hiding process, the original ransomware APK file was fed as an input to VirusTotal engines. Subsequently, the scanning process was performed again on the stego frame (PNG file) resulting after applying the hiding process. Finally, the stego frame was merged with the original video frames and the entire infected video (AVI file) was scanned. The results of applying ransomware scanning analysis on the three files (APK, PNG, AVI) are illustrated in Figure 2. As Figure 2a demonstrates, performing the scanning on the original ransomware APK alerted 34 out of 64 antivirus engines about the malicious behavior of this APK. However, after applying the proposed approach, none of the antivirus engines were able to detect the concealed ransomware within the cover frame (Figure 2b). Additionally, the hidden ransomware within the infected video was also undetectable by any of the VirusTotal engines (Figure 2c).

### 4.5. Original/Extracted Ransomware Analysis

The comparison between the original and extracted ransomware APK files was performed on two levels, APK-level and code-level. The APK-level comparison was performed using version 1.0 of Apkcompare tool (https://github.com/linsea/ApkCompare accessed on 1 August 2021). The tool takes as an input the two APK files and generates a file listing the differences between the provided APK’s. However, in our tests, the generated comparison files were empty, indicating identical APKs. On the other hand, in order to implement a code-level analysis, the ransomware APK files were decompiled using Apktool (https://ibotpeaches.github.io/Apktool/ accessed on 1 August 2021)) to retrieve the original source code. Therefore, decompiling the original and the extracted ransomware APK files resulted in retrieving the manifest files and the small files of each APK, respectively. Subsequently, the retrieved source code files were compared using the Diffchecker tool (https://www.diffchecker.com accessed on 1 August 2021). The comparison analysis of the source code files reveals no difference between the original and the extracted ransomware files.This proves the optimal extraction of the embedded encrypted ransomware from the HEVC streams.

Furthermore, to ensure the integrity of the proposed approach, the hash function SHA256 has been applied on the ransomware samples before the embedding and after performing the extraction algorithm. Table 8 shows the digest of the original ransomware samples matches the digest of the resulting ransomware succeeding the extraction process. Thus, it can be concluded that the ransomware samples were recovered completely and correctly.

As can be observed from the above results, the mission of anti-steganography technology against our approach is very challenging due to the following reasons:

- The difficulty of knowing the original cover media, mainly when it is in the format of video streaming. The attacker can utilize any trending video to hide ransomware there. It is not easy and maybe not be possible for the anti-steganography to be aware of the original video to detect the presence of the hidden data.
- The randomness of choosing the video frames and bits to embed the ransomware. This makes it even more difficult to recognize where the ransomware is hidden.
- Even if the above two conditions are satisfied and the anti-steganography techniques manage to locate the hidden ransomware within a video stream, the extracted data will be encrypted, and the key is needed to decrypt it and then analyze it, which is also very challenging as only the attacker knows the key.

Therefore, our proposed approach is robust against the most common antivirus software even though they are equipped with up-to-date malware detection solutions.

## 5. Conclusions

This paper demonstrated that the available and modern ransomware detection systems have critical weaknesses because they do not consider the ransomware hiding possibility. Additionally, they do not check how to detect, extract, and analyze the concealed ransomware apps inside multimedia data. In this work, an efficient hybrid crypto-steganography approach has been introduced for ransomware hiding based on AES encryption and LSB-based HEVC steganography. Consequently, this paper has exploited the video steganography process and tested its performance to hide encrypted ransomware apps inside high-resolution video streams. Comprehensive objective and subjective assessment metrics were utilized extensively to examine the proposed hybrid approach compared to related research works. In addition, various HEVC streams with different resolutions were tested to explore the hiding performance of various ransomware apps with diverse sizes. The experimental analysis disclosed that the proposed hybrid approach succeeded in hiding ransomware and bypassing all security and quality tests. Thus, it was concluded and noticed from the obtained results that the key advantages of the proposed crypto-steganography approach are (1) building a multi-level security framework for ransomware hiding using both encryption and steganography processes, (2) ensuring the ransomware privacy to avoid detection possibility even when screened by standard antivirus engines, (3) achieving strong embedding performance by preserving the quality and the main characteristics of the cover video stream after ransomware concealing, (4) performing the embedding process in a randomness manner to mitigate attack possibility, (5) choosing the best pixel location to embed the ransomware apps to maintain video imperceptibility, (6) extracting the complete hidden ransomware samples successfully, (7) verifying the integrity of the transmitted ransomware by calculating and comparing the hash values of the ransomware; before the embedding and after the extraction, and (8) achieving superior performance in terms of all investigated subjective and objective tests.

The proposed crypto-steganography approach can be used in different offensive and defensive cybersecurity applications, such as (a) achieving high confidentiality of the secret data, whether benign or malicious, (b) malware/ransomware distribution through online communications, (c) Android mobile systems, (d) security of multimedia communication systems, (e) malware detection applications, and (f) the confidentiality of Android IoT data.

As future work, various lightweight encryption algorithms compatible with IoT devices’ resources can be investigated to encrypt the ransomware samples before embedding them in the multimedia content. Moreover, different multimedia files (e.g., audio and image) may be utilized as cover media. Furthermore, we intend to develop deep learning algorithms to check if they could detect the concealed ransomware samples inside the video streams. Additionally, more steganalysis techniques can be examined to investigate the robustness performance of the proposed crypto-steganography approach. These steganalysis techniques assume that the original video used to hide the ransomware is known to the detectors. Additionally, the detector needs to break the key/algorithm used to encrypt the ransomware. Then the ordinary scanning approaches used by different antivirus systems can be applied.

## Figures and Tables

**Table 1:** Resolution of the tested video streams.

HEVC Stream	Resolution
GTFly	3840 × 2160
Microworld	2560 × 1600
Shark	1920 × 1088
Kimono	1280 × 720
Balloon	640 × 480

**Table 2:** Size of the tested ransomware samples.

Test Sample	Size
Ransomware 1	1.036 MB
Ransomware 2	734 KB
Ransomware 3	390 KB
Ransomware 4	171 KB
Ransomware 5	60 KB

**Table 3:** The employed visual objective quality assessment metrics.

Metric	Definition and Recommended Value (Cover/Stego)	Optimal Value
Mean Square Error (MSE) [42]	Calculate error percentage between cover and stego frames. Low MSE value is recommended.	0
Peak Signal-to-Noise Ratio (PSNR) (dB) [43]	Measure the quality of the stego frame referring to the cover frame. High value is recommended.	>20 dB
Signal-to-Noise Ratio (SNR) (dB) [44]	Estimate the average power of the cover frame referring to the one of the difference between the stego and cover frames. High value is recommended.	>20 dB
Weighted Signal-to-Noise Ratio (WSNR) (dB) [45]	Determine the amount of average weighted power of the cover frame referring to the one of the difference between the stego and cover frames. High value is recommended.	>20 dB
Noise Quality Measure (NQM) (dB) [46]	Estimate the amount of distortion in terms of local luminance, contrast perception, and frequency shift between the stego and cover video frames. High value is recommended.	>20 dB
Structural Content (SC) [47]	Estimate the amount of power for the cover frame referring to the amount of power for the stego frame. Low value is recommended.	1
Maximum Difference (MD) [48]	Estimate the difference between the stego and cover frames. Low value is recommended.	<5
Normalized Absolute Error (NAE) [43]	Estimate the absolute difference value between the stego and cover video frames referring to the absolute value of the cover frame. Low value is recommended.	0
Laplacian Mean Square Error (LMSE) [48]	Determine the edges difference between stego and cover frames. Low value is recommended.	0
Structural Similarity Index (SSIM) [49]	Assess the visual structural similarity between the stego and cover frames. High value is recommended.	1
Multi-Scale SSIM index (MS-SSIM) [50]	Evaluate the multi-scale structural similarity between the stego and cover frames. High value is recommended.	1
Feature Similarity Index (FSIM) [51]	Estimate the feature similarity between the stego and cover frames. High value is recommended.	1
Universal Quality Index (UQI) [52]	Evaluate the universal not local similarity in terms of correlation/contrast/luminance quantities between the stego and cover video frames. High value is recommended.	1
Normalized Cross Correlation (NK) [53]	Compare the stego frame with the reference cover frame. High value is recommended.	1
Average Difference (AD) [54]	Estimate the average difference between cover and stego frames. Low value is recommended.	0
Pixel-based Visual Information Fidelity (VIFP) [55]	Compare the similarity information of pixels content of the stego and cover frames. High value is recommended.	1
Bit Error Rate (BER) [41]	Estimate the pixel error rate between the stego and cover frames. Low value is recommended.	0
Edge detection ratio (EDR) [48]	Determine the Gaussian Laplacian difference at the edges. Low value is recommended.	0
Entropy (E) [48]	Estimate the amount of information in the video frame. It is recommended to get a lower value of difference entropy between the cover/stego frames (ΔE).	(ΔE) = 0

**Table 4:** The employed objective quality assessment metrics.

HEVC Stream	Cover Frame	Stego Frame	Difference Frame
Balloon (Frame 5) (Ransomware 5)	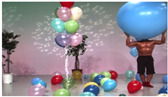 (Entropy = 7.4830)	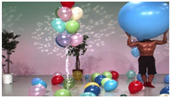 (Entropy = 7.4934, CPU time = 1.3749 s)	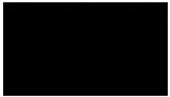 (Entropy = 0.5758 and BER = 0)
Kimono (Frame 10) (Ransomware 4)	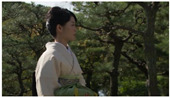 (Entropy = 6.8912)	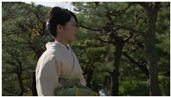 (Entropy = 6.8924, CPU time = 1.7652 s)	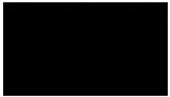 (Entropy = 0.4649 and BER = 0)
Shark (Frame 15) (Ransomware 3)	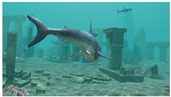 (Entropy = 7.2341)	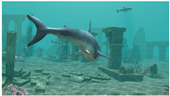 (Entropy = 7.2383, CPU time = 2.4907 s)	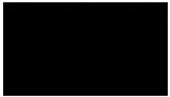 (Entropy = 0.5324 and BER = 0)
Microworld (Frame 20) (Ransomware 2)	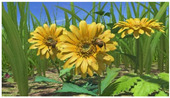 (Entropy = 7.6303)	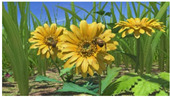 (Entropy = 7.6318, CPU time = 3.1012 s)	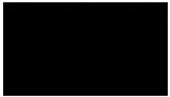 (Entropy = 0.4992 and BER = 0)
GTFly (Frame 25) (Ransomware 1)	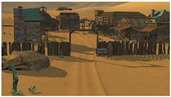 (Entropy = 6.9411)	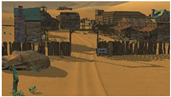 (Entropy = 6.9429, CPU time = 3.8416 s)	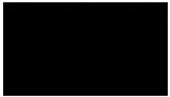 (Entropy = 0.3746 and BER = 0)

**Table 5:** Histogram outcomes of the tested HEVC frames in case of using different ransomware samples.

HEVC Stream	Cover Frame	Stego Frame	Difference Frame
Balloon (Frame 5) (Ransomware 5)	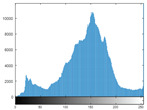	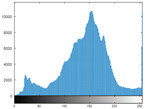	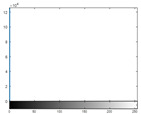
Kimono (Frame 10) (Ransomware 4)	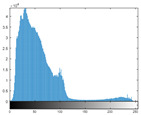	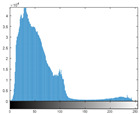	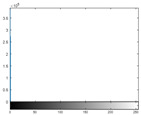
Shark (Frame 15) (Ransomware 3)	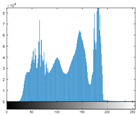	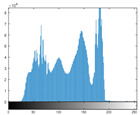	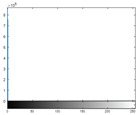
Microworld (Frame 20) (Ransomware 2)	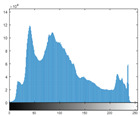	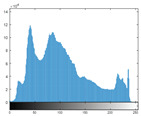	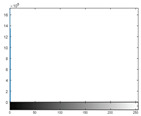
GTFly (Frame 25) (Ransomware 1)	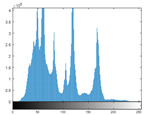	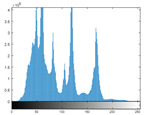	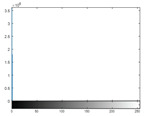

**Table 6:** Gaussian Laplacian edges of the tested cover and stego HEVC frames in case of using different ransomware samples.

HEVC Stream	Cover Frame	Stego Frame
Balloon (Frame 5) (Ransomware 5)	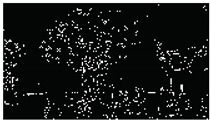	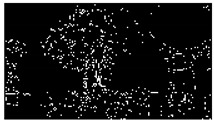 (EDR = 0)
Kimono (Frame 10) (Ransomware 4)	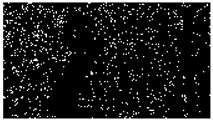	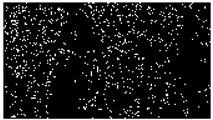 (EDR = 0)
Shark (Frame 15) (Ransomware 3)	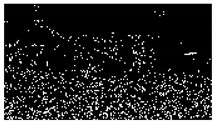	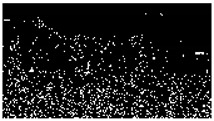 (EDR = 0)
Microworld (Frame 20) (Ransomware 2)	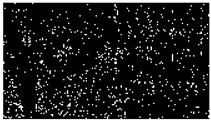	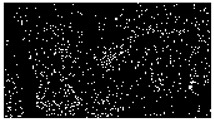 (EDR = 0)
GTFly (Frame 25) (Ransomware 1)	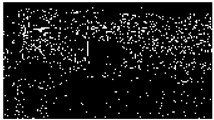	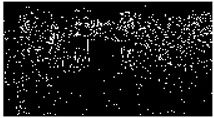 (EDR = 0)

**Table 7:** Comparison results of objective quality assessment of tested video streams.

Metric	Approach	Balloon	Kimono	Shark	Microworld	GTFly
MSE	This work	0.1765	0.0972	0.0955	0.0795	0.0473
	[58]	0.4657	0.0984	0.1172	0.0874	0.1136
	[38]	0.3625	0.1038	0.1273	0.0817	0.1208
	[39]	0.2976	0.0992	0.0983	0.0901	0.0892
PSNR (dB)	This work	55.6638	58.2528	58.3326	59.1297	61.3848
	[58]	51.45	58.1309	57.4431	58.7163	57.5758
	[38]	52.718	56.3341	57.6728	57.9571	57.9781
	[39]	53.6103	57.4051	57.0187	58.5707	58.6273
SNR (dB)	This work	34.5556	36.9058	37.3517	38.2439	40.6255
	[58]	29.0289	36.8942	35.8633	35.8633	35.651
	[38]	30.8278	34.8641	35.8934	36.8679	36.8997
	[39]	32.2409	35.9647	36.7639	37.6817	37.9534
WSNR (dB)	This work	62.207	59.2711	64.5086	66.1695	67.0911
	[58]	50.5367	55.203	56.6973	60.5513	54.9348
	[38]	50.0891	55.3927	55.9894	60.7813	55.8397
	[39]	51.4831	56.7161	57.4387	61.4637	56.5974
NQM (dB)	This work	41.0328	47.1413	33.7515	42.4614	42.2109
	[58]	33.7358	46.694	31.0799	40.9216	35.9405
	[38]	33.7352	45.6712	31.8274	40.7618	36.0081
	[39]	34.9856	46.8674	32.1127	41.2064	37.1346
SC	This work	1.0001	1.0002	1	1	1
	[58]	1.0006	1.0004	1.0001	1	1.5002
	[38]	1.0008	1.0006	1.0005	1.0003	1.1201
	[39]	1.0004	1.0003	1.0003	1.0002	1.081
MD	This work	2	1	1	1	1
	[58]	6	4	3	3	5
	[38]	4	3	2	3	4
	[39]	3	2	2	2	3
NAE	This work	0.0013	0.0016	0.00075	0.00065	0.00049
	[58]	0.0018	0.0019	0.00077	0.00067	0.00083
	[38]	0.0017	0.002	0.0024	0.0071	0.0073
	[39]	0.0015	0.0018	0.002	0.0068	0.0067
LMSE	This work	0.0065	0.0037	0.0037	0.0011	0.002
	[58]	0.0081	0.0058	0.0051	0.0079	0.0043
	[38]	0.0079	0.0053	0.0049	0.0057	0.0067
	[39]	0.0072	0.0049	0.0043	0.0034	0.0054
SSIM	This work	0.9986	0.9996	0.9994	0.9997	0.9997
	[58]	0.9971	0.9993	0.9992	0.9995	0.9995
	[38]	0.9973	0.9991	0.9987	0.9991	0.9991
	[39]	0.9979	0.9994	0.9989	0.9994	0.9993
MSSIM	This work	0.9983	0.9997	0.9985	0.9998	0.9999
	[58]	0.995	0.9995	0.9967	0.9997	0.9997
	[38]	0.9974	0.999	0.9971	0.9989	0.999
	[39]	0.9979	0.9992	0.9979	0.9991	0.9995
FSIM	This work	0.9999	1	0.9999	1	1
	[58]	0.9986	0.9997	0.9996	0.9998	0.9996
	[38]	0.9991	0.999	0.9971	0.9989	0.9993
	[39]	0.9994	0.9992	0.9979	0.9991	0.9995
UQI	This work	1	0.9997	1	1	1
	[58]	1	0.9995	1	0.9998	0.9998
	[38]	0.9997	0.9992	0.9997	0.9995	0.9993
	[39]	1	0.9994	0.9999	0.9997	0.9996
NK	This work	0.9999	0.9999	1	1	1
	[58]	0.9997	0.9998	0.9999	0.9998	0.9998
	[38]	0.9994	0.9993	0.9995	0.9992	0.9996
	[39]	0.9996	0.9996	0.9997	0.9996	0.9997
AD	This work	0.0022	0.0056	−0.0016	−0.003	−0.0031
	[58]	0.0548	0.006	0.0064	0.0022	0.0076
	[38]	0.0427	0.0089	0.0071	0.0064	0.0083
	[39]	0.0292	0.0071	0.0059	0.0048	0.0067
VIFP	This work	0.9994	0.9998	0.9997	0.9998	0.9997
	[58]	0.9972	0.9997	0.9988	0.9993	0.9988
	[38]	0.9979	0.9991	0.9991	0.9991	0.9987
	[39]	0.9984	0.9995	0.9993	0.9994	0.9991
(ΔE)	This work	0.5758	0.4649	0.5324	0.4992	0.3746
	[58]	0.6725	0.4922	0.5794	0.5375	0.4242
	[38]	0.6913	0.4937	0.6108	0.5798	0.5221
	[39]	0.6149	0.4706	0.6098	0.5578	0.497

**Table 8:** Hash-256 values of ransomware samples before and after implementing the extraction algorithm.

Ransomware Sample	Original	Extracted
Ransomware 5	0b996d80d1c721c5ddc5991d45a 84b27906619524a9a7518d12 47dfafda3f15a	0b996d80d1c721c5ddc5991d45a 84b27906619524a9a7518d12 47dfafda3f15a
Ransomware 4	4c82672ee77ac1229e8de9a33ec22 33d650ee3b2133cc30569822 adae50bc0ed	4c82672ee77ac1229e8de9a33ec22 33d650ee3b2133cc30569822 adae50bc0ed
Ransomware 3	3fb373be488bd20f5055e413824b4 5d2ab56e1949c58a867a9a6 8118d119c97e	3fb373be488bd20f5055e413824b4 5d2ab56e1949c58a867a9a6 8118d119c97e
Ransomware 2	01b7fafdd11276012f5480c7ea4215 22c287bda88b756d77521157 65bcbb2b19	01b7fafdd11276012f5480c7ea4215 22c287bda88b756d77521157 65bcbb2b19
Ransomware 1	72f87a4199d3a295d45ca7eda532d 41842687813f4c6ea22a716 bec66d4f5288	72f87a4199d3a295d45ca7eda532d 41842687813f4c6ea22a716 bec66d4f5288

## Data Availability

No new data were created or analyzed in this study. Data sharing is not applicable to this article.
